# Explainable AI for gait speed analysis from multimodal data fusion

**DOI:** 10.1371/journal.pone.0341067

**Published:** 2026-02-27

**Authors:** Abdullah Alharthi, Abdulrahman Al Ayidh, Ahmed Alqurashi, Mohammed Alammar, Shoaib Shahriar Mohammad, Houssem Bouchekara, Yusuf Abubakar Sha’aban, Turki Essa Alharbi

**Affiliations:** 1 Department of Electrical Engineering, King Khalid University, Abha, Saudi Arabia; 2 Department of Electrical Engineering, Umm Al Qura University, Makkah, Saudi Arabia; 3 Department of Electrical Engineering, University of Hafr Al Batin, Hafr Al Batin, Saudi Arabia; 4 Department of Computer and Software Engineering, Embry-Riddle Aeronautical University, Prescott, Arizona, United States of America; 5 Center for International Studies, Massachusetts Institute of Technology, Cambridge, Massachusetts, United States of America,; 6 Department of Electrical Engineering, College of Engineering, Taif University, Taif, Saudi Arabia; Sunway University, MALAYSIA

## Abstract

Gait speed analysis is vital for applications in healthcare, rehabilitation, human-robot interaction, and autonomous systems, necessitating robust methods to address its complexity. This study introduces an advanced framework for gait speed classification through multimodal data fusion and deep learning, optimized using Layer-wise Relevance Propagation (LRP). Utilizing a publicly available 4 datasets of 50 injury-free adults walking at varying speeds, we integrate data from full-body motion capture, electromyography (EMG), and force plates to capture detailed gait dynamics. We propose two novel architectures: a hybrid Convolutional Neural Network with Long Short-Term Memory (CNN+LSTM) and a Multi-stream CNN, benchmarked against Temporal Convolutional Networks (TCNs), Transformer neural networks, Gated Recurrent Units (GRU) and statistical classifiers (e.g., Linear Discriminant Analysis, Quadratic Discriminant Analysis, Support Vector Machines). All models in this study were trained, validated and tested using three distinct strategies: experiment 1 (sample-based splitting – 10-fold cross-validation), experiment 2 (10-fold cross-validation, subject-based splitting – 5 subjects for testing), and experiment 3 (5-fold cross-validation, subject-based splitting – 10 subjects for testing). Multi-stream Quads CNN is the best-performing model, achieving the highest F1 scores across all experiments (96.6% ± 1.4% experiment 1, 96.2% ± 1.6% experiment 2, 95.9% ± 0.9% experiment 3 with top performance for all experiments 98% ± 0.6%, outperforming traditional approaches. LRP is applied to evaluate feature relevance within the model, enabling the removal of critical features to verify robustness. Comprehensive assessments, including ROC curves, confusion matrices, t-test, and perturbation analyses, validate the model’s enhanced performance and durability. By combining multimodal fusion to overcome single-sensor constraints with LRP-driven feature optimization, this work provides a highly accurate and resilient tool for gait analysis, with significant potentials.

## 1. Introduction

The walking style or pattern of humans, referred to as human gait, is a complex motor behavior critical to understanding human movement dynamics across multiple disciplines. It is useful in biomechanics, neuroscience, and rehabilitation sciences to assess mobility, diagnose neurological disorders, and develop therapeutic interventions. Detailed gait analysis provides insights that inform applications such as human–vehicle interaction (HVI) and human-robot interaction (HRI) [1]. In both HVI and HRI domains, for example, precise gait speed measurements allow for the proper prediction of human movement patterns, which can be explored for safer sharing of spaces between autonomous vehicles, robots, and humans [2,3]. Gait speed analysis is important in early disease diagnosis and fall detection in the elderly, among other emerging uses such as biometric authentication systems [1,2]. Gate speed is a valuable biomarker for cognitive decline and a tool for monitoring the progression of neurodegenerative diseases and predicting the onset of functional dependence in the elderly [4–7].

The performance of gait speed analysis is highly dependent on both data quality and quantity. Additionally, traditional analysis methods that rely solely on manual visual observations or a single sensor modality are inherently limited in capturing the intricate details of human gait. Consequently, there has been a growing emphasis on multi‐modal data fusion. This technique integrates data from multiple sensors to construct an enhanced representation of human movement patterns [1]. Moreover, the accessibility of multi-modal fusion techniques is witnessing further enhancements due to the adoption of advanced artificial intelligence (AI) techniques.

However, these developments have also introduced significant challenges in data acquisition. Assembling large, internally consistent multi‐modal gait databases is an arduous task. Despite this challenge, there are many publicly available human gait databases. Therefore, developing classification tools that can be trained on publicly available databases is a crucial first step toward improving gait speed analysis [2]. This paper presents a novel approach that exploits the spatiotemporal characteristics of human gait by leveraging multi‐modal data fusion in conjunction with a publicly available database.

Key contributions of this work include:

- The introduction of two innovative architectures: a composite Convolutional Neural Network with Long Short-Term Memory (hybrid CNN+LSTM) and a Multi-stream Convolutional Neural Network for robust data processing.
- The implementation of statistical machine learning techniques, including Linear Discriminant Analysis, Quadratic Discriminant Analysis, and Support Vector Machines for control, benchmarking, and reference.
- A comprehensive evaluation of the proposed machine learning models through the presentation of confusion matrices, receiver operating characteristic (ROC) curves, Mathew’s correlation coefficient (MCC) curves, and detailed analyses of model accuracy and loss.
- The integration of explainable AI (XAI) methods to systematically examine how classification accuracy is affected by distinct input variables, with XAI analyzers being employed to compare the impact of various markers on the overall classification process.

The subsequent sections of the paper are organized as follows. Section 2 gives an extensive literature appraisal of the dynamics of human gait and the application of explainable AI in gait analysis. Section 3 details the data acquisition methods, preprocessing techniques, and the proposed machine learning models, along with a discussion of the markers used for data management and the XAI methodology. Section 4 presents experimental results, focusing on gait classification performance and the comparative analysis of various XAI analyzers. The findings of this work are discussed in Section 5, and the paper is finally concluded in Section 6.

## 2. Related work

Understanding human gait dynamics is essential for numerous applications in healthcare, biomechanics, and robotics [1]. In healthcare, gait patterns and dynamics have been recognized as both diagnostic and prognostic tools. For example, high gait variability is associated with certain conditions such as Parkinson’s disease, lower limb injury, and aging [8] However, healthy individuals also exhibit considerable gait variability due to fluctuations in walking speed and other factors. For instance, in a study, young female adults demonstrated the lowest gait variability when walking at approximately 10% above their preferred speeds [9]. Hence, making inferences based on gait speeds is often not straightforward. Consequently, the research community has extensively studied human gait recognition and dynamics [1].

However, capturing the complexity of gait across different walking speeds presents a significant challenge [10]. In this work, we investigate the fusion of multimodal gait spatiotemporal data to provide a comprehensive analysis across varying speeds. By integrating data from diverse sources such as inertial sensors, video analysis, and pressure-sensitive platforms, we aim to offer a holistic perspective on gait dynamics. This synthesis enhances the accuracy of gait analysis and facilitates the development of robust algorithms for gait classification, rehabilitation, and assistive devices [11].

Recent advances in multimodal sensor technologies have enabled the collection of diverse data sets that offer a comprehensive view of human gait while substantially enhancing the accuracy and robustness of gait speed classification [12,13]. In particular, data augmentation using multiple sensor modalities can effectively mitigate the limitations of individual sensors, thereby improving classification performance [13,14]. Moreover, the integration of advanced data fusion techniques, machine learning algorithms, and statistical analysis has further refined the precision of gait analysis [11]. Several studies have investigated the fusion of electromyography (EMG) data with either inertial measurement unit (IMU) or electroencephalography (EEG) signals to enhance gait analysis through multimodal sensing [11]. For instance, in [15], a configuration incorporating 12 EEG channels and 2 EMG sensors was employed to analyze gait in patients with Parkinson’s disease. The investigation revealed that key features, such as walking speed, stride time, step time, and EEG power (analyzed via independent component analysis), were significantly reduced in patients with Parkinson’s disease and older individuals compared to healthy, younger subjects. Furthermore, these groups exhibited a shortened swing phase, underscoring the impact of neurodegenerative conditions on gait dynamics.

Current gait recognition systems predominantly leverage deep learning methods that have evolved from earlier approaches such as template matching and conventional machine learning [12]. Modern architectures, including recurrent neural networks (RNN) [16], gated recurrent units (GRU) [17], and long short-term memory (LSTM) networks [18], demonstrate significant improvements in automatically extracting and classifying gait features from wearable inertial sensor data. For instance, the sequential convolution LSTM network described in [12] effectively mitigates the high time-series and spatial information loss that often plagues standalone CNN and LSTM approaches. Despite their impressive performance, these methods are frequently criticized for their “black-box” nature, which can hinder clinical adoption. Consequently, explainable artificial intelligence (XAI) techniques have been increasingly integrated into gait analysis to elucidate the underlying decision processes, enhancing trust among patients and practitioners.

Recent studies underscore the potential of XAI in the realm of gait analysis using multimodal sensor data. Kang et al. [19] and Mishra et al. [20] employed XAI to optimize sensor placement, reduce system complexity in human-machine interfaces, and analyze patient gait features using knee-ankle-foot orthoses. These investigations highlight the role of XAI in enhancing both the performance and interpretability of gait analysis systems. Moreover, Katmah et al. [11] emphasized the significance of multimodal sensor fusion, particularly through the incorporation of electromyographic (EMG) signals, in improving gait analysis accuracy, while Slijepcevic et al. [21] demonstrated that XAI methods can effectively elucidate class-specific characteristics learned by machine learning models in clinical settings.

In a recent study, Kim et al. [22] integrated XAI with wearable sensor-based gait analysis to identify patients with osteopenia and sarcopenia during daily activities. Their work revealed that specific gait features, such as stride variability and joint kinematics, are critical indicators of musculoskeletal health deterioration. Building on these insights, our work employs XAI techniques to investigate and interpret the key gait features that influence gait speed classification using multimodal sensor data. By enhancing model transparency and interpretability, our approach aims to facilitate the development of robust, real-time gait analysis systems for clinical decision support.

The integration of explainable AI (XAI) with machine learning (ML) has been explored to automate gait analysis for foot disorders, achieving high accuracy while identifying key features of clinical relevance [23]. The proposed ML pipeline, interpreted using LIME, shows considerable promise in aiding both diagnosis and surgical planning for foot disorders. Additionally, an automated method for classifying pathological gait patterns based on three-dimensional ground reaction force (GRF) data has been developed; this method effectively distinguishes healthy individuals from those with conditions such as cerebral palsy (CP) or multiple sclerosis (MS). Feature selection techniques have further enhanced classification accuracy, with improvements from 85% to 95% observed when using the nearest neighbor classifier (NNC) and artificial neural networks (ANN) [24]. Furthermore, the application of XAI methods, particularly Layer-wise Relevance Propagation (LRP), has been investigated to increase transparency in automated clinical gait classification [21]. In addition, ML methods for classifying CP-related gait patterns were studied [25], revealing that traditional models outperform deep neural networks. Notably, when using kinematic data, specifically sagittal knee and ankle angles, the random forest classifier achieved an accuracy of 93.4%, underscoring the critical role of explainability in ML for clinical applications.

## 3. Materials and methods

Human gait speed is a critical biomechanical parameter that quantitatively indicates underlying physiological and neurological conditions. It is widely recognized as a primary metric for assessing disease severity, functional mobility, and overall health, thus playing an essential role in rehabilitation and clinical evaluation. Although gait speed might appear to be a straightforward measure, its determination arises from a complex interplay among biomechanical forces, environmental influences, and neuromuscular control.

This study presents a comprehensive approach to classifying gait speed by integrating multidimensional data acquired from full-body motion capture, electromyography (EMG), and force plates. By combining these modalities, we construct a robust and detailed representation of human movement that enables extracting salient gait features. We employ hybrid deep learning architecture, merging Convolutional Neural Networks (CNNs) with Long Short-Term Memory (LSTM) networks to identify features that differentiate between distinct speed classes automatically. Moreover, we incorporate Explainable AI (XAI) techniques to interpret model predictions, thereby increasing the transparency and clinical relevance of the gait classification process.

**Fig 1:**
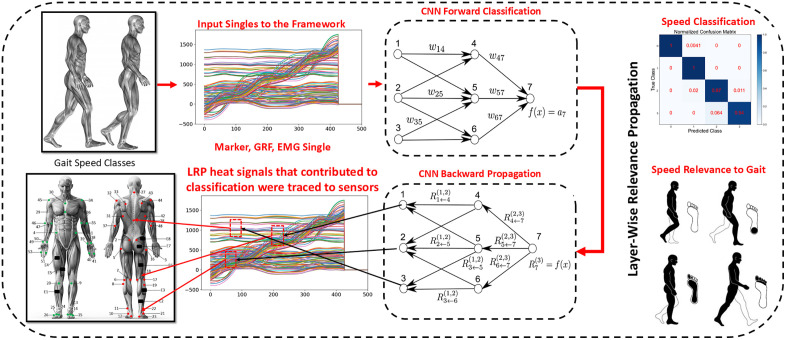
Gait speed analysis framework. Gait signals classification is traced to the sensor location using LRP for speed relevance to gait.

Central to our investigation is gait classification into various speed categories, as illustrated in Fig 1. These classifications not only facilitate the mapping of the dynamic spectrum of human movement but also enable a more profound analysis through XAI methods. Specifically, by applying XAI, we elucidate which features among the myriad of captured signals most strongly correlate with gait speed variations. This inquiry leads to several key research questions:

1. How does gait speed vary across different physiological conditions, and what are the underlying features that signify these variations?
2. What are the principal biomechanical and neuromuscular components that differentiate healthy and impaired gait states?
3. How do external forces, quantified through ground reaction forces, interact with internal processes such as muscle activity to determine gait speed?
4. What insights can XAI provide regarding the hierarchical importance of features influencing gait speed, and how might these insights enhance diagnostic and rehabilitative strategies?

Our methodology, detailed in the subsequent sections, encompasses data collection, preprocessing, the design of our deep learning architecture, and the integration of XAI for interpretability. This holistic approach not only ensures reliable gait speed classification but also yields critical insights into the biomechanical and clinical factors that govern human locomotion. Ultimately, by analyzing variations in gait speed and movement patterns, our work aims to advance the scientific understanding of human mobility and contribute to improved health assessments and rehabilitation practices.

### 3.1. Data and data-processing

#### 3.1.1. Overview.

This study utilized a publicly available multimodal human gait dataset [26,27] collected from 50 healthy adults, including 26 males and 24 females (mean age: 37.0 ± 13.6 years; mean height: 1.74 ± 0.09 m; mean weight: 71.0 ± 12.3 kg). “The study was approved by the institutional medical ethic committee of the Rehazenter and follows the recommendations of the declaration of Helsinki. The participants gave their informed consent to participate in the study” [26]. All participants were verified to be free from upper or lower limb injuries in the six months prior to data collection and had not undergone any surgeries on these limbs in the past two years.

Gait recordings were obtained as participants walked along a straight, level walkway under five controlled speed conditions: very slow (0–0.4 m/s), slow (0.4–0.8 m/s), moderate (0.8–1.2 m/s), self-selected natural speed, and fast preferred speed [26]. Raw data were stored in the C3D file format [27], a standard widely used in biomechanics that synchronizes multimodal measurements.

#### 3.1.2. Data description.

**Fig 2:**
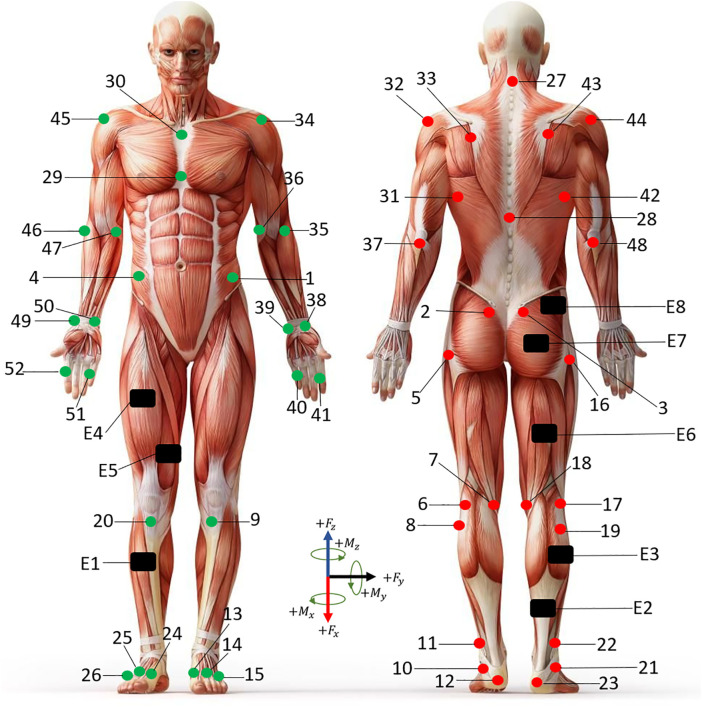
52 reflective skin markers were positioned on the subjects through anatomical palpation (see Table 1). Green markers illustrate the front side, while red markers indicate the back. The markers are numbered according to their order in the spatial domain. EMG signals recorded from probes on the right leg muscles are represented in black (E1–E8).

**Fig 3:**
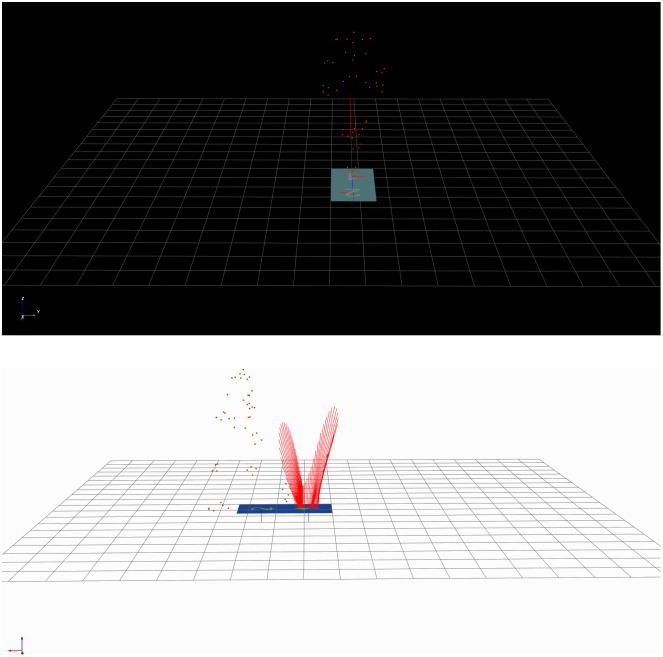
MOKKA-Motion Kinematic & Kinetic Analyzer reflective markers and force plates. a: front view, b: side view.

Each trial contained two complete gait cycles (left and right), resulting in a total of 1142 ⨯ 4 with total number of samples 4568 sample recordings across all participants. The datasets captures multiple complementary modalities:

- Markers (Trajectories of Reflective Markers): 3D Cartesian coordinates of 52 (see Table 1) reflective markers placed on anatomical landmarks, sampled at 100 Hz, capturing precise spatiotemporal body motion (Figs 2 and 3).
- GRF (Ground Reaction Forces): Measured at 1500 Hz using force plates, centre of pressure coordinates, 3D ground reaction force, 3D ground reaction moment (Fig 3).
- EMG (Electromyography): Eight surface electrodes (E1–E8) sampled at 1500 Hz, providing neuromuscular activity across gait phases.
- F&M (Forces and Moments): Force applied by the foot on force plate and Moment applied by the foot on force plate.

**Table 1:** 52 Markers labels.

#	Label	Description	#	Label	Description
1	L_IAS	Left anterior–superior iliac spine coordinates	27	R_IPS	Right posterior–superior iliac spine coordinates
2	L_IPS	Left posterior–superior iliac spine coordinates	28	R_IAS	Right anterior–superior iliac spine coordinates
3	L_FTC	Left greater trochanter coordinates	29	R_FTC	Right greater trochanter coordinates
4	L_FLE	Left lateral femoral epicondyle coordinates	30	R_FLE	Right lateral femoral epicondyle coordinates
5	L_FME	Left medial femoral epicondyle coordinates	31	R_FME	Right medial femoral epicondyle coordinates
6	L_FAX	Left fibula head coordinates	32	R_FAX	Right fibula head coordinates
7	L_TTC	Left tibial tuberosity coordinates	33	R_TTC	Right tibial tuberosity coordinates
8	L_FAL	Left lateral tibial malleolus coordinates	34	R_FAL	Right lateral tibial malleolus coordinates
9	L_TAM	Left medial tibial malleolus coordinates	35	R_TAM	Right medial tibial malleolus coordinates
10	L_FCC	Left posterior calcaneus coordinates	36	R_FCC	Right posterior calcaneus coordinates
11	L_FM1	Left 1st metatarsal head coordinates	37	R_FM1	Right 1st metatarsal head coordinates
12	L_FM2	Left 2nd metatarsal head coordinates	38	R_FM2	Right 2nd metatarsal head coordinates
13	L_FM5	Left 5th metatarsal head coordinates	39	R_FM5	Right 5th metatarsal head coordinates
14	CV7	7th cervical vertebra coordinates	40	TV10	Spinous process of the 10th thoracic vertebra coordinates
15	SXS	Suprasternal notch coordinates	41	SJN	Xiphoid process coordinates
16	L_SIA	Left acromial tip coordinates	42	R_SIA	Right acromial tip coordinates
17	L_SRS	Left spine root coordinates	43	R_SRS	Right spine root coordinates
18	L_SAA	Left acromial angle coordinates	44	R_SAA	Right acromial angle coordinates
19	L_SAE	Left acromial edge coordinates	45	R_SAE	Right acromial edge coordinates
20	L_HLE	Left lateral humerus epicondyle coordinates	46	R_HLE	Right lateral humerus epicondyle coordinates
21	L_HME	Left medial humerus epicondyle coordinates	47	R_HME	Right medial humerus epicondyle coordinates
22	L_UOA	Apex of the left olecranon coordinates	48	R_UOA	Apex of the right olecranon coordinates
23	L_RSP	Left radius styloid process coordinates	49	R_RSP	Right radius styloid process coordinates
24	L_UHE	Left ulnar styloid process coordinates	50	R_UHE	Right ulnar styloid process coordinates
25	L_HM2	Left head of the 2nd metacarpus coordinates	51	R_HM2	Right head of the 2nd metacarpus coordinates
26	L_HM5	Left head of the 5th metacarpus coordinates	52	R_HM5	Right head of the 5th metacarpus coordinates

These modalities together provide a detailed representation of kinematic, kinetic, and neuromuscular aspects of human gait. Table 2 summarizes the datasets for all participants.

**Table 2:** Forces and Moments; N: Number of samples is 1142.

Data type	Temporal	Dim.	Spatial
3D Markers (N)`	585	3	156
3D GRF (N)	585	3	24
F&M (N)	8985	3	12
EMG (N)	8985	1	8

Temporal indicates the number of frames per trial, Dim. indicates the dimensionality of each data point (3 for 3D data, 1 for EMG), Spatial indicates the number of channels/features per modality.

Table 2. Datasets for 50 Participants. Markers: Trajectories Reflective Markers; GRF: Ground Reaction Force; EMG: Electromyography; F&M.

#### 3.1.3. Data processing.

All raw C3D recordings were processed using MOKKA (Motion Kinematic & Kinetic Analyzer) software [28]. MOKKA was employed to visualize recordings, extract features, and convert data into structured formats suitable for computational analysis. Specifically, MOKKA facilitated the extraction of spatiotemporal data for each modality according to the following dimensions: Due to differences in walking speed, sequence lengths varied across participants. All trials were zero-padded to match the longest sequence, ensuring consistent input dimensions across samples and facilitating effective training and comparative analyses.

#### 3.1.4. Preparation for machine learning.

Before model training, features from all modalities were standardized to zero mean and unit variance to ensure comparability across measurements with different scales. Training in all experiments was conducted with early stopping using a patience of 20 epochs to prevent overfitting while retaining the best model weights. Details of training procedures and experimental results are presented in Section 4.

The dataset was then organized into three experimental strategies:

1. **Experiment 1 – Sample-Based Splitting (10-Fold Cross-Validation with Reshuffling):** Data was randomly divided into training, testing, and validation sets, and a 10-fold cross-validation procedure was applied. In each fold, the data was reshuffled using a fixed random seed to reduce sampling bias and ensure reproducibility.
2. **Experiment 2 – Subject-Based Splitting (5 Subjects for Testing with Reshuffling):** Data was split at the subject level to maintain independence between training and testing. In a 5-fold subject-wise cross-validation scheme, 5 subjects were reserved for testing in each fold, while the remaining participants were used for training and validation. The subjects were reshuffled in each fold using a fixed random seed.
3. **Experiment 3 – Subject-Based Splitting (10 Subjects for Testing with Reshuffling):** This setup was identical to Experiment 2, except that 10 subjects were reserved for testing in each fold. A 5-fold subject-wise cross-validation procedure was applied, with subjects reshuffled in each fold using a fixed random seed.

### 3.2. Machine learning methods

Human gait is a complex and multifaceted biomechanical process that provides valuable insights into a wide range of clinical conditions and rehabilitative outcomes. Capturing subtle variations in gait speed requires sophisticated analytical approaches capable of handling high-dimensional, multi-source data generated during movement, including full-body motion sensors, EMG recordings, and force plates. To test these hypotheses, we employed a combination of statistical analyses and deep learning techniques to comprehensively analyze the intricate spatiotemporal patterns inherent in gait dynamics.

In this study, we leverage multiple advanced deep learning models to comprehensively analyze gait data. Convolutional Neural Networks (CNNs) are employed to capture spatial and local temporal correlations between body segments and muscle activations, enabling automatic feature extraction without extensive manual engineering. Temporal Convolutional Networks (TCNs) complement CNNs by modeling longer-range temporal dependencies across gait cycles, while Gated Recurrent Unit (GRU) networks and Long Short-Term Memory (LSTM) are used to learn sequential dependencies in both forward and backward directions, capturing dynamic gait patterns over time. Additionally, Transformer algorithms are incorporated to exploit attention mechanisms that highlight salient features and interactions across time steps, enhancing the model’s ability to detect subtle changes in gait speed.

To ensure robust evaluation and prevent information leakage between layers, the algorithms were trained and tested using three experimental strategies. The first experiment used a sample-based splitting approach with 10-fold cross-validation, reshuffling the data in each fold to reduce sampling bias and ensure reproducibility. The second experiment employed a subject-based splitting scheme, reserving five subjects per fold for testing while training and validation were performed on the remaining participants, with reshuffling applied to maintain independence. The third experiment extended this subject-based design by holding out ten subjects per fold for testing, again using reshuffling to avoid overlapping. This systematic training strategy ensures sufficient data coverage, validates model generalizability, and prevents inadvertent information leakage among the machine learning layers. All codes are available in github (https://github.com/Abdullah00Alharthi/Explainable-AI-for-Gait-Speed-Analysis-from-Mul-timodal-Data-Fusion).

Moreover, to complement model performance with interpretability, explainable artificial intelligence (XAI) methods are integrated alongside the deep learning models. XAI techniques allow for the validation of learned features against established biomechanical principles, quantify the importance of different variables, and provide insight into the spatiotemporal and physiological patterns that drive changes in gait speed. This multi-model, multi-strategy framework ensures both robust prediction accuracy and interpretability, laying the foundation for the subsequent analysis of gait dynamics.

#### 3.2.1. Multi-source fusion CNN algorithms.

**Fig 4:**
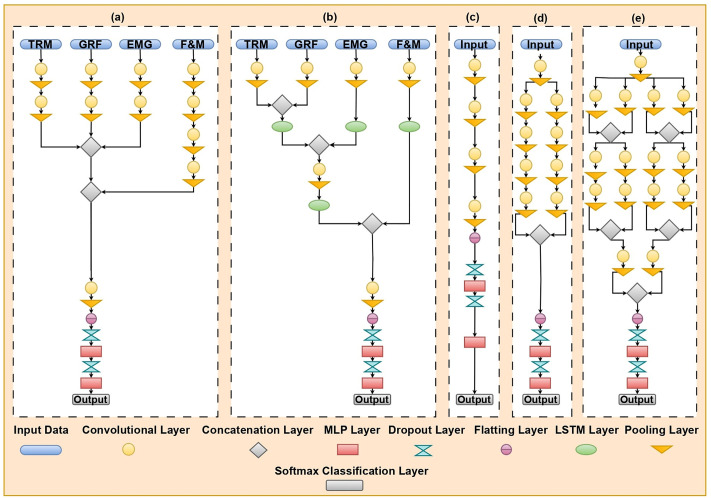
CNN architecture. a) Multi-Source Fusion CNN, b) Hybrid CNN+LSTM network, c) Single CNN, d) Dual CNN, e) Quads CNN.

Multi-stream CNN networks have been proposed as part of this research, building upon an extensive body of previous work that has established foundational concepts and methodologies in the field [23,29]. The Multi-stream CNN architecture, as illustrated in Fig 4, is specifically designed to integrate multiple streams of data at the feature level effectively. This integration is achieved through weight sharing during both the training and validation stages, allowing the network to learn more generalized features from the diverse data inputs. The model can better capture complex interactions among the different data streams by focusing on feature-level fusion, enhancing its representational power. The performance of this network was evaluated on a test set consisting of randomly selected subjects, specifically focusing on a cohort of 10 individuals. This careful selection process ensures that the results are robust and can be generalized to a broader population. The proposed architecture aims to enhance the model’s ability to capture intricate patterns in the data, ultimately leading to improved performance in various applications.

#### 3.2.2. Hybrid CNN+LSTM network algorithms.

The human gait data is inherently structured in spatial and temporal dimensions, making it particularly suitable for advanced analytical techniques. To leverage this unique characteristic, we propose a hybrid CNN+LSTM network architecture. In this model, the convolutional layers extract meaningful patterns from the input data, effectively identifying local features representing different gait aspects. Following the convolutional layers, LSTM cells assess which extracted features should be retained and passed on through the network layers. This decision-making process is crucial, especially considering the fusion of four different data types, as it allows the model to maintain relevant information while discarding noise or less significant features. The stacked layers of this network architecture are illustrated in Fig 4, providing a clear visual representation of the interplay between the convolutional and LSTM components. At the top of the network, a Multi-Layer Perceptron (MLP) is incorporated, utilizing a SoftMax activation function for classification purposes. This final layer enables the model to produce output probabilities for each class, facilitating effective decision-making based on the processed gait features. The combination of CNNs and LSTMs in this architecture is designed to enhance the model’s ability to capture both spatial and temporal dynamics, ultimately leading to improved performance in gait analysis applications.

#### 3.2.3. Single-source feature fusion CNN algorithms.

We design a CNN framework that leverages different processing streams for feature extraction to achieve feature fusion using single-stream and multi-stream CNN architectures for training a single data source from a single sensing modality. As depicted in Fig 4, the single-stream CNN architecture integrates multiple processing streams by utilizing weight sharing during training and validation stages. This approach allows the network to capture generalized features more effectively from a single data input, promoting robust learning across the entire dataset.

In this architecture, feature-level fusion enables the model to learn complex interactions within the data, enhancing its representational capacity. A test set comprising randomly selected individuals (specifically a cohort of 10) was used to validate the model’s robustness, ensuring the results were generalized. This architecture is designed to detect intricate patterns in the data, resulting in improved performance across various applications.

#### 3.2.4. Temporal Convolutional Network (TCN) algorithms.

Multi-layer Temporal Convolutional Network (TCN) is utilized to classify gait sequences by capturing both short-term and long-term temporal dependencies across motion features. The input to the model consists of multi-dimensional gait sequences represented as (timesteps,features), which are first standardized and one-hot encoded to normalize feature distributions and prepare labels for classification. Each sequence is processed through a stack of four TCN blocks with increasing numbers of filters (12, 24, 48, and 96), where each block performs dilated causal convolutions to model temporal dependencies at multiple scales. Batch normalization follows each block to stabilize and accelerate training, while dropout is applied to prevent overfitting. The outputs of the final TCN block are flattened through fully connected dense layers with 100 units and additional dropout before producing class probabilities via a SoftMax activation.

#### 3.2.5. Gated recurrent unit network algorithms.

Multi-dimensional gait sequences are analyzed using a Gated Recurrent Unit (GRU) network, which processes temporal information in both forward and backward directions to better capture the dynamics of human movement. The network consists of three stacked bidirectional GRU layers with 128, 256, and 256 units, each followed by dropout and batch normalization to improve training stability and prevent overfitting. These recurrent layers extract temporal patterns from the sequences, integrating information from past and future timesteps. The output is then passed through a series of fully connected dense layers (512, 256, and 128 units) that further combine the temporal features and prepare them for classification. The final layer uses softmax activation to predict gait class probabilities. The model was trained and tested in three separate data experiments to evaluate its robustness, using 5-fold cross-validation to assess performance. Results demonstrate that this architecture effectively captures complex temporal dependencies in gait data, providing accurate multi-class classification.

#### 3.2.6. Transformer algorithms.

In this study, gait sequences are classified using a Transformer-based neural network, designed to capture temporal dependencies across motion features. The input to the model consists of multi-dimensional gait sequences represented as (timesteps, features), which are first standardized and one-hot encoded to normalize feature distributions and prepare labels for classification. Each input sequence is passed through a stack of Transformer encoder blocks, each comprising a multi-head self-attention mechanism and a feed-forward network [30]. The multi-head attention enables the model to compute relationships between all timesteps within the sequence, producing context-aware representations of each gait feature, while residual connections and layer normalization stabilize training. The feed-forward layers further enhance the feature representation at each timestep. Following the Transformer encoders, a Global Average Pooling layer aggregates information across the temporal dimension, producing a single feature vector for each sequence. This vector is then processed through fully connected dense layers with dropout for regularization before producing final class probabilities using a softmax activation. The model is trained and evaluated in 3 experiments with different validation schemes to ensure that sequences from the same subject do not appear in both training and test sets, thus preventing data leakage. This architecture has demonstrated robust classification performance in prior applications of Transformer models for time-series data [31].

#### 3.2.7. Statistical classifiers algorithms.

Traditional ML methods, such as Linear Discriminant Analysis (LDA), Quadratic Discriminant Analysis (QDA) and Support Vector Machines (SVM), are suggested as baseline and benchmark models for gait classification. These models use a single feature set for each classification task, meaning no fusion is involved. The data fed into these methods is reshaped into samples to create a 1D vector.

#### 3.2.8. Explainable AI algorithms.

To provide interpretability, we employ layer-wise relevance propagation [32–34]. LRP is a reverse propagation technique that identifies the most important features in an input vector for neural networks to generate predictions. The prediction is backward propagated through a redistribution process in which the relevance of the neurons is reallocated across the network via backpropagation until it reaches the input layer. Generally, LRP produces a heatmap on the original signal, highlighting regions with the most significant influence on the model’s prediction, such as areas exhibiting the most variation between classes. It should be noted that a neural network consists of several layers of neurons, where neurons are activated, as described in [32].


$$\begin{array}{c} a_{k}=\sigma (\sum {\mathrm{\backslash nolimits}}_{j}^{n}a_{j}\omega_{jk\;}+b_{k}) \end{array}$$ (1)

In (1), $a_{k}$ represents the activation of the neuron while $a_{j}$ denotes the neuron activation in the previous layer during the forward pass. $\omega_{jk}$ refers to the weight received by neuron *k* from neuron *j* in the preceding layer, and $b_{k}$ is the bias. The sum is calculated across every of the j-th neurons linked to the k-th neuron. σ is a monotonically rising, nonlinear activation function. CNN learns the biases, activation, and weights through supervised training. In the training phase, the model computes the output $f_{c}(x)$ via forward pass, while backpropagation uses the model errors to adjust the parameters $\left(\omega_{jk\;}+\;b_{k}\right)$ are adjusted through backpropagation using the model’s error. We rely on categorical cross-entropy for this process for our computation [34].

The LRP method breaks down the CNN output of the gait class prediction function $f_{c}$ for any input $x_{i}$ to obtain a relevance score R for the i-th neuron received from $R_{j}$ representing the relevance of the j-th neuron in the preceding layer, which is passed down from $R_{k}$, the relevance of the k-th neuron in the subsequent layer, where the relevance conservation principle is maintained as:


$$\begin{array}{c} \sum {\mathrm{\backslash nolimits}}_{i}R_{i⟵j}=\;\sum {\mathrm{\backslash nolimits}}_{j}R_{j⟵k}=\sum {\mathrm{\backslash nolimits}}_{k}R_{k}=f_{c}(x) \end{array}$$ (2)

The LRP process begins at the CNN final layer following the removal of the *Softmax* layer. In this procedure, a specific gait class c is selected for LRP, while the remaining classes are excluded. During unpooling, backpropagation in the pooling layer is performed by tracing the signal back to the neuron whose activation was computed during the forward propagation step. To illustrate, imagine a single output neuron i in one of the layers of the model, which is assigned a relevance score $R_{j}$ from a neuron j, in a lower-layer, or the final output of the model (class c). The relevance scores are distributed across the interconnected neurons throughout the network layers based on how much each input signal $x_{i}$ contributes, using the activation function (calculated during the forward propagation and updated via backpropagation during training) of neuron j. This neuron will retain a specific relevance score determined by its activation and transfer its value to the following neurons in the reverse direction. Ultimately, the method produces relevance scores for each sensor input at distinct time intervals. The resulting scores generate a heatmap, where areas with the highest relevance scores at certain time points highlight the features that played the most prominent role in the model’s classification. Other propagation rules, like the (αβ-rule) also exists [32].

The significance of accurately classified gait patterns was determined by establishing logical variables, and a relevance score was then allocated to each input variable. LRP calculates the relationship between variables and the predicted model results, then normalizes the association patterns derived from LRP to their respective maximum values for comparison. Next, the average of all relevant patterns was computed, and any errors were corrected. The corrected average was smoothed, where the current value was given a 50% weight, and the preceding and subsequent points were each assigned 25%. During the smoothing process, the weighted values were adjusted so that their total equaled 1, and repeating the process approximated a Gaussian filter. These steps were repeated three times to achieve the desired result. Lastly, the smoothed correlation pattern was rescaled from 0 (no correlation) to 1 (maximum correlation). Since the input variables are gathered in the time domain, where adjacent values are interdependent, the variation in the relevance score is minimized through smoothing. To examine the impact of different variables on classification accuracy, all variables were ranked based on their correlation, and the top 200 variables with the highest relevance scores were selected for further explanation and analysis of the gait pattern.

#### 3.2.9. Explainable AI decomposition evolution.

To assess the effectiveness of classification, we use a step-by-step process that evaluates how the class-specific information in the gait data sample (quantified by a function f) diminishes as we systematically remove information from different regions of the input signal x. This process, known as region perturbation, involves altering specific areas of the gait data sample to observe how the class representation is affected [22]. Unlike the method in [35] that focuses on flipping binary pixel states (single-pixel perturbation), the [33] approach generalizes this by allowing perturbations on broader regions, such as local windows, using methods like randomization ∙A heatmap, in this context, is defined as an ordered set of locations within the gait data sample, represented $as\;O=(r_{\text{1}}r_{\text{2}},...,r_{l}$) where each location $r_{p}$ specifies a position on a grid of pixels (e.g., horizontal and vertical coordinates). The sequence of locations can be defined manually or determined through a heat mapping function, $h_{p}=H(x,f,r_{p})$, which typically relies on a class-specific discriminant function f. The values $\left\{h_{p}\right\}$ indicate the relevance of each location $r_{p}$ in representing the class within data. The sequence is organized so that for all indices in O, if i<j then $\left(H\left(x,f,r_{i}\right)>\;H\left(x,f,r_{i}\right)\right)$. Consequently, the most relevant regions for the classifier function f appear at the beginning of the sequence, while the least relevant areas are placed towards the end.

This perturbation approach follows this ordered sequence of locations, where we first remove information from the most relevant areas (referred to as “Most Relevant First” or MoRF).


$$x_{MoRF}^{\left(\text{0}\right)}=x$$ (3)

$$\forall \text{1}\leq k\leq L:\;x_{MoRF}^{\left(k\right)}=g\left(x_{MoRF}^{\left(k-\text{1}\right)},r_{i}\right)$$ (4)

The process is described by a recursive formula, where the function g removes data $x_{MoRF}^{\left(k-\text{1}\right)}$ at a specific location $r_{k}$ in the data, either as a data point or a local area. Throughout this paper, we use g to replace data points within a 7·7 neighborhood around $r_{k}$ with randomly sampled values from a uniform distribution.

When comparing heatmaps with a fixed perturbation function $g(x,r_{k})$, we focus primarily on the highly relevant areas, disregarding the ranking of less important regions. The key metric here is the area over the MoRF perturbation curve (AOPC), calculated as:


$$\begin{array}{c} AOPC=\;\frac{\text{1}}{L+\text{1}}\sum {\mathrm{\backslash nolimits}}_{K}^{L}\left(f\left(x_{MoRF}^{\left(\text{0}\right)}\right)-f\left(x_{MoRF}^{\left(k\right)}\right)\right).p(x) \end{array}$$ (5)

Where (∙) $p_{\left(x\right)}$ denotes the average across all gait data samples in the dataset. Ordering places the most sensitive regions first results in a sharper decline in the MoRF curve, producing a higher AOPC value.

## 4. Experiments and results

This study addresses philosophical and theoretical questions about human gait speed through controlled experiments designed to classify various gait speeds. In particular, the investigation delves into the biomechanical and neuromuscular factors contributing to gait variability. Data is collected from a set of modality sensors, including full-body motion capture systems, electromyography (EMG) sensors, and force plates, which can provide comprehensive spatiotemporal data capturing the intricate dynamics of human gait across a range of speeds.

The primary approach involved training a series of machine learning algorithms such as Convolutional Neural Networks (CNNs), Temporal Convolutional Networks (TCNs), Gated Recurrent Unit (GRU) networks and Long Short-Term Memory (LSTM) networks and transformer algorithms to analyze the high-dimensional sensor data. The machine learning algorithms architecture incorporated multiple layers to discern spatial relationships across sensor inputs. This setup enabled the models to extract meaningful features like muscle activity patterns, joint angles, ground reaction forces, and other critical elements for precise gait speed analysis from the raw data.

### 4.1. Experiments and models training

All models were trained and tested using Python 3.11.5, Keras 2.15.0, and TensorFlow 2.15.0. The primary training was conducted on a system equipped with an Intel(R) Core(TM) i7-1065G7 CPU @ 1.30GHz (up to 1.50GHz), an NVIDIA GeForce MX330 GPU (4 GB), and Intel(R) Iris(R) Plus Graphics (128 MB). To accelerate the training of computationally demanding architectures such as the Transformer, CNN+LSTM, and Temporal Convolutional Network (TCN), additional experiments were executed on Google Colab utilizing GPU acceleration.

To ensure the reliability and generalization of the results while preventing data leakage, all models in this study were trained using three distinct strategies. Experiment 1 (Sample-Based Splitting – 10-Fold Cross-Validation with Reshuffling) involved randomly dividing the dataset into training, validation, and testing subsets, followed by a 10-fold cross-validation procedure in which the data was reshuffled using a fixed random seed to minimize sampling bias and ensure reproducibility. Experiment 2 (Subject-Based Splitting – 5 Subjects for Testing with Reshuffling) maintained independence between subjects by performing the split at the subject level, applying a 5-fold subject-wise cross-validation strategy where five subjects were reserved for testing in each fold while the remaining subjects were used for training and validation; the subject distribution was reshuffled in every fold using a fixed random seed. Experiment 3 (Subject-Based Splitting – 10 Subjects for Testing with Reshuffling) followed the same setup as Experiment 2 but reserved ten subjects for testing in each fold, employing a 5-fold subject-wise cross-validation scheme with subjects reshuffled in each iteration using a fixed random seed to ensure consistent evaluation and eliminate data leakage. All machine learning models were optimized using the Adam optimizer, chosen for its adaptive learning rate and robustness in handling sparse gradients and noisy data, which enhances convergence stability. Early stopping was also implemented to prevent overfitting and reduce unnecessary computation once model performance plateaued, with a patience value of 20 epochs — meaning training would stop if no improvement was observed for 20 consecutive epochs. Each fold was trained for up to 1000 epochs, allowing sufficient iterations for convergence across the various architectures.

### 4.2. Gait classification

**Fig 5:**
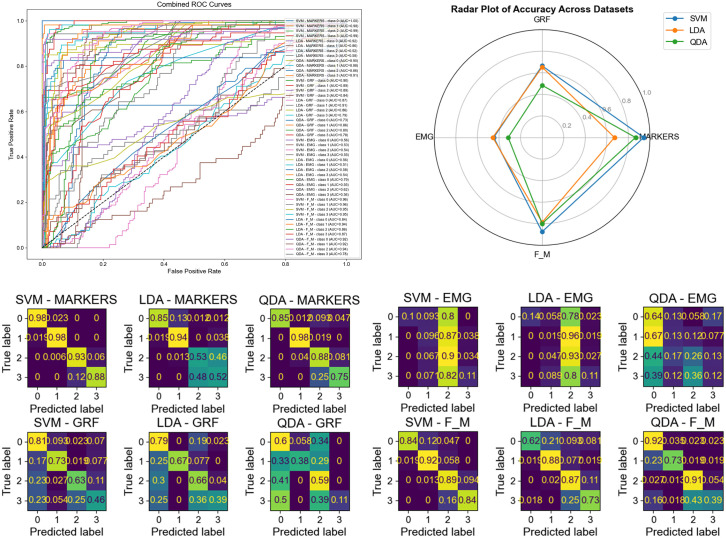
Gait 4 speeds classification experiment 1 top performance models Using GRF, EMG, Markers, and F&M Data with (Linear Discriminant Analysis, Quadratic Discriminant Analysis, SVM). The plot displays a model ROC curve, radar spider chart, and confusion matrices. Top performance for Markers data using SVM Accuracy: F1 Score 94%. a: ROC Curve, b: Spider (Radar) Chart, c: Confusion Matrix.

**Fig 6:**
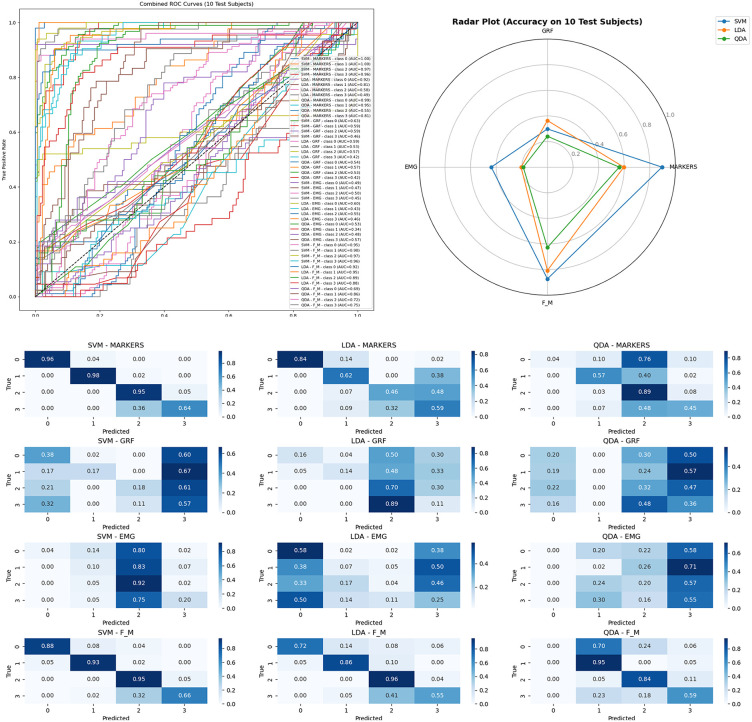
Gait 4 speeds classification experiment 3 top performance model Using GRF, EMG, Markers, and F&M Data with (Linear Discriminant Analysis, Quadratic Discriminant Analysis, SVM). The plot displays a model ROC curve, radar spider chart, and confusion matrices. a: ROC Curve, b: Spider (Radar) Chart, c: Confusion Matrix.

The dataset used in this study consists of gait recordings from multiple subjects walking at different speeds, designed to evaluate how effectively various classification algorithms can distinguish between distinct gait patterns. The data were partitioned into training and testing subsets under controlled experimental setups to ensure fair model comparison and robust evaluation. Figs 5 and 6 present the comparative performance of three classifiers—Support Vector Machine (SVM), Linear Discriminant Analysis (LDA), and Quadratic Discriminant Analysis (QDA)—applied to four data modalities: Ground Reaction Force (GRF), Electromyography (EMG), Markers, and combined Force & Marker (F&M) data. Specifically, Fig 5 reports the testing outcomes for Experiment 1 (Sample-Based Splitting) Fig 5a: ROC Curve, Fig 5b: Spider (Radar) Chart, Fig 5c: Confusion Matrix, while Fig 6a, (b), (c) presents the testing results for Experiment 3 (Subject-Based Splitting with 10 subjects for testing).

Each Fig includes multiple performance visualizations to provide a comprehensive analysis. The Receiver Operating Characteristic (ROC) curves display the performance across four distinct gait classes (Class 0 through Class 3) by plotting the True Positive Rate (TPR) against the False Positive Rate (FPR) across a range of classification thresholds. These curves provide insight into each model’s discriminative capability for differentiating gait patterns at varying confidence levels. The radar plots in Figs 5 and 6 complement the ROC curves by illustrating the models’ relative performance across different evaluation metrics—such as accuracy, F1 score, precision, and recall—across all classes and data modalities. Additionally, the confusion matrices provide a class-by-class visualization of prediction accuracy, highlighting instances of misclassification and offering deeper insight into which gait classes the models tend to confuse. Together, these visualizations present a holistic overview of model strengths, weaknesses, and inter-class performance consistency.

From the results, the SVM classifier consistently outperformed the other methods across all experiments and data types. It achieved an overall accuracy of 0.941 and an F1 score of 0.941, with AUC values ranging between 0.98 and 1.00 across all classes, indicating strong separability and high discriminative power. The QDA model exhibited moderate performance, achieving an accuracy of 0.865 and an F1 score of 0.866, with AUC values ranging from 0.86 to 0.91, showing its ability to capture some nonlinear relationships but with less stability across classes. In contrast, the LDA model underperformed, particularly in Classes 1 and 2, with an accuracy of 0.670 and an F1 score of 0.687, reflecting its limitation in handling overlapping or nonlinear data distributions.

In Table 3, the SVM model achieved the most consistent and superior F1 scores across all experiments and data modalities. For the Markers data, SVM reached 94.1%, 89.6%, and 89.1% F1 scores in Experiments 1, 2, and 3, respectively, while the combined F&M data yielded 87%, 84.2%, and 87%. Comparatively, LDA and QDA demonstrated lower performance, particularly when applied to GRF and EMG data, which contain higher signal variability and noise. The radar plots further emphasize this performance gap, as SVM consistently dominates in every metric, forming a more uniform and extended polygon across the radar axes, while LDA and QDA show irregular and constricted shapes, reflecting inconsistent performance. The confusion matrices reinforce this trend, with SVM showing a more pronounced diagonal dominance, indicating precise classification across classes, while LDA and QDA exhibit higher off-diagonal misclassifications.

**Table 3:** Classification F1-Score to Classify Gait Speed.

Model type	DATA	Experiment 1: F1 score	Experiment 2: F1 score	Experiment 3: F1 score
SVM	GRF	66.3% ± 3.6%	31.3% ± 6.6%	30.1% ± 8.4%
	EMG	35.7% ± 3.1%	35% ± 4.2%	34.1% ± 7.3%
	Markers	94.1% ± 1.7%	89.6% ± 3.5%	89.1% ± 2.7%
	F&M	87% ± 2.3%	84.2% ± 3.7%	87% ± 2.1%
LDA	GRF	68.6% ± 5%	29.7% ± 1.9%	33.6% ± 4.8%
	EMG	36.1% ± 3.5%	35.1% ± 2.09%	16.5% ± 10.7%
	Markers	67.4% ± 5.5%	65.2% ± 3.5%	61.9% ± 7.8%
	F&M	78% ± 6.8%	79.5% ± 3.6%	80% ± 5.6%
QDA	GRF	46.9% ± 4.9%	24.3% ± 3.5%	22.7% ± 12.6%
	EMG	30.2% ± 3.3%	22.3% ± 5.9%	16% ± 3.6%
	Markers	58.2% ± 9.6%	46.8% ± 9.7%	49.7% ± 3.9%
	F&M	77% ± 2.4%	50.7% ± 8.3%	55.8% ± 4.4%
Single CNN	GRF	81.2% ± 4.4%	56.5% ± 1%	63.6% ± 1.4%
	EMG	84.1% ± 3.2%	94% ± 1.8%	90% ± 3.1%
	Markers	95.4% ± 2.6%	92.8% ± 4.54%	91% ± 4.2%
	F&M	89% ± 1.8%	86.2% ± 1.2%	87.9% ± 2.8%
Dual CNN	GRF	88.4% ± 4.4%	64.8% ± 13.6	63.09% ± 10.8%
	EMG	94.3% ± 1.6%	91.3% ± 6.8	90.5% ± 2.1%
	Markers	95.3% ± 1.1%	92.7% ± 3.52%	91.3% ± 1.61%
	F&M	93% ± 1.75%	90.09% ± 3.77%	89.2% ± 1.2
Quads CNN	GRF	82.4% ± 1.4%	81.6% ± 2.3%	81.3% ± 1.3%
	EMG	93.3% ± 1.0%	91.5% ± 1.3%	92.4% ± 1.4%
	Markers	**96.6% ± 1.4%**	**96.2% ± 1.6%**	**95.9% ± 0.9%**
	F&M	93% ± 1.3%	91% ± 2.0%	89% ± 1.8%
Multi-Source Fusion CNN	All DATA	96% ± 0.3%	93% ± 1.3%	90% ± 2.8%
Hybrid CNN+LSTM	All DATA	95% ± 0.6%	90% ± 2.6%	91.2% ± 1.7%
Temporal CNN (TCN)	GRF	64.7% ± 4.4%	63.72% ± 4.4%	61.1% ± 12.07
	EMG	37.06% ± 7.4%	34.1% ± 8.6%	37.06% ± 7.4%
	Markers	93.3% ± 1.68%	94% ± 1.1%	93.5% ± 2.1%
	F&M	91% ± 2.34%	88.3% ± 2.6%	80% ± 1.9%
Transformer	GRF	44.08% ± 8.4%	41.6% ± 3.7%	39.7% ± 6.5%
	EMG	61.1% ± 5.6%	62.3% ± 4.1%	59.1% ± 1%
	Markers	93.77% ± 1.09%	89.8% ± 2.1%	89.5% ± 2.6%
	F&M	87% ± 2.08%	89.4% ± 2.65%	81% ± 2.87%
GRU	GRF	43.18% ± 7.4%	44.6% ± 4.6%	37.6% ± 7.4%
	EMG	51.4% ± 8.56%	54.7% ± 10.01%	53.6% ± 9.8%
	Markers	85% ± 1.35%	87% ± 2.6%	81% ± 1.2%
	F&M	75% ± 2.35%	77% ± 3.2%	79.3% ± 1.9%

Overall, the results across ROC curves, radar plots, and confusion matrices confirm that the SVM classifier provides the most reliable and generalizable performance across different data sources and experimental settings. Its stability and adaptability make it a robust baseline for multi-source gait classification, demonstrating superior capability in capturing complex biomechanical variations across subjects and walking speeds.

In addition to the classical classifiers, several CNN-based and hybrid deep learning models were developed to categorize gait speeds, as illustrated in Fig 4. All models were trained using the backpropagation algorithm with the Adam optimizer and a multi-class cross-entropy loss function. To ensure robustness and generalizability, each model was evaluated using k-fold cross-validation, and key metrics including accuracy, precision, recall, and F1 score were computed for each fold. Table 3 summarizes the F1 scores of all models across different sensor modalities (GRF, EMG, markers, F&M) and three experimental setups.

Among the traditional classifiers, SVM achieved its highest performance with marker data, attaining an F1 score of 0.941 in Experiment 1, but exhibited poor performance with EMG (≈0.357) and GRF (≈0.667). LDA and QDA showed comparatively low F1 scores across all modalities, particularly for EMG and GRF data, underperforming relative to SVM and the deep learning models.

The CNN-based models demonstrated substantial improvements over traditional methods. The Quads CNN consistently achieved the highest F1 scores, reaching 0.966 for markers in Experiment 1, and maintained strong performance in Experiments 2 (0.962) and 3 (0.959), though its performance on GRF was lower (0.824, 0.816, 0.813 across Experiments 1–3). The Dual CNN also maintained strong performance for EMG and marker data (F1 ≈ 0.943–0.953 for Exp 1, 0.913–0.927 for Exp 2, 0.905–0.913 for Exp 3), but showed reduced performance on GRF (0.884, 0.648, 0.631). Single CNN models achieved F1 scores of 0.954 for markers and 0.921 for EMG in Experiment 1, with moderate declines in Experiments 2 and 3. Multi-Source Fusion CNN and Hybrid CNN+LSTM models consistently performed well across all modalities, with F1 scores exceeding 0.95 for Experiment 1 and remaining above 0.90 in Experiments 2 and 3.

Temporal models including Temporal CNN (TCN), Transformer, and GRU exhibited more variable performance across experiments. TCN achieved high F1 scores for marker data (0.933, 0.940, 0.935 for Experiments 1–3) but underperformed on GRF (0.647, 0.637, 0.611) and EMG (0.371, 0.341, 0.371). Transformer models performed well on markers (0.938, 0.898, 0.895) but had low scores on GRF (0.441, 0.416, 0.397) and moderate performance on EMG (0.611, 0.623, 0.591). GRU models were less consistent, achieving moderate performance on markers (0.85, 0.87, 0.812) and F&M (0.75, 0.772, 0.793), but lower F1 scores for GRF (0.432, 0.446, 0.376) and EMG (0.514, 0.547, 0.536) across Experiments 1–3. These results suggest that while temporal and recurrent models can capture sequential dependencies, their performance varies significantly depending on sensor modality and experimental setup.

**Fig 7:**
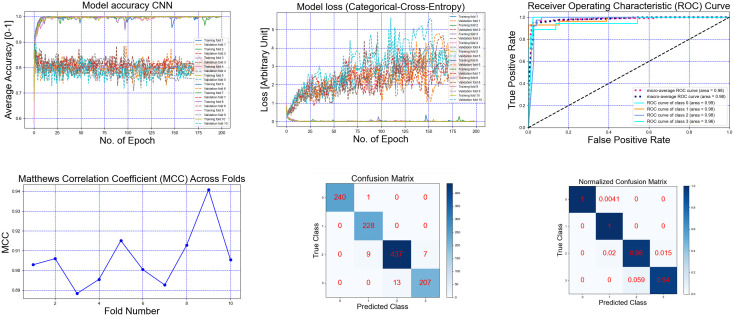
Gait 4 speeds classification with Using EMG Data and Quads CNN top performance model experiment 1. (a) Accuracy across folds, (b) model loss across folds, (c) ROC curve, (d) Matthews Correlation Coefficient (MCC) across folds, (e) Confusion matrix and (f) Normalized confusion matrix.

**Fig 8:**
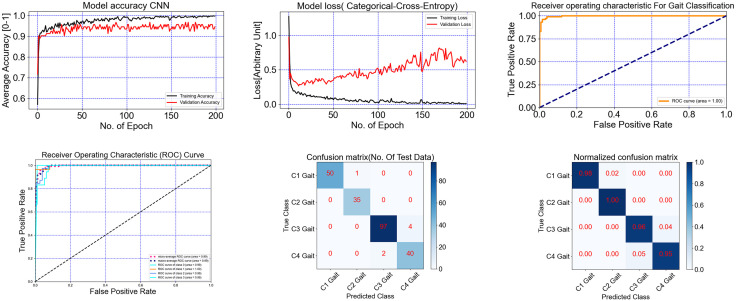
Gait 4 speeds classification with Using Marker Data and Dual CNN, precision, recall, and f1-score 97% top performance model experiment 1. (a) The model accuracy, (b) The model training loss, (c) ROC for Gait classification, (d) ROC for multi-class Gait, (e) The confusion matrix, and (f) Normalized confusion matrix.

Figs 7 and 8 illustrate detailed model evaluation. For instance, the Quads CNN trained on EMG data (Experiment 1) achieved high training accuracy, as shown in Fig 7a, though validation accuracy fluctuated, suggesting opportunities for further optimization. Training and validation loss curves (Fig 7b) demonstrated effective learning, while ROC curves (Fig 7c) yielded AUC values from 0.96 to 0.99. Matthews Correlation Coefficient (MCC) values (Fig 7d) supported prediction robustness, particularly for imbalanced data. Confusion matrices (Figs 7e, f) showed near-zero off-diagonal entries, confirming accurate class prediction. Similarly, evaluation curves for the Dual CNN model on marker data (Fig 8) demonstrated high training accuracy and low loss, while ROC curves and confusion matrices confirmed reliable classification across all gait speeds. Overall, deep learning models, particularly Quads CNN, Dual CNN, Multi-Source Fusion CNN, and Hybrid CNN+LSTM, consistently outperformed traditional classifiers across all experiments. Temporal and recurrent models (TCN, Transformer, GRU) effectively captured sequential patterns but showed reduced robustness on GRF and EMG data, highlighting the importance of modality-specific considerations and cross-validation design for gait speed classification.

### 4.3. T-Test analysis

#### 4.3.1. Model-to-model analysis.

**Fig 9:**
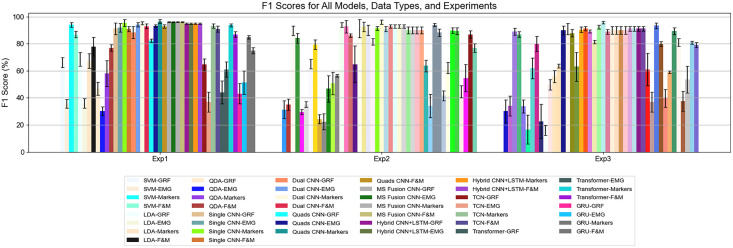
F1 scores for all models based on the data type and experiment 1 to 3.

**Fig 10:**
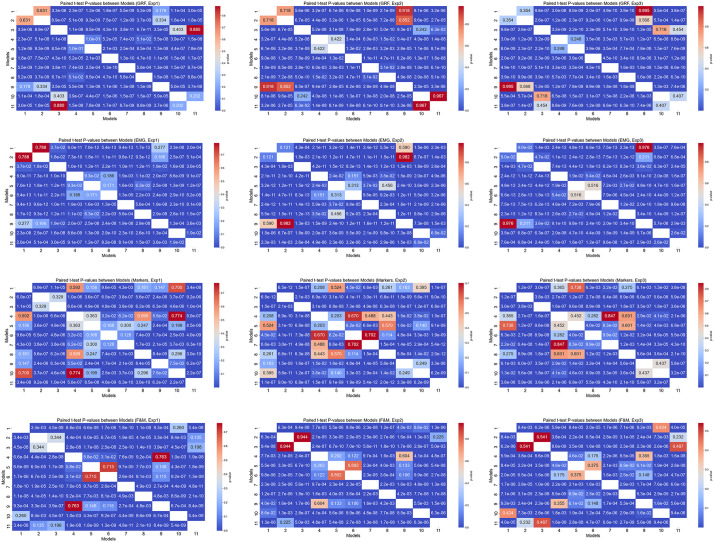
Heatmap values t-test and P-values between models: 1. SVM, 2. LDA, 3. QDA, 4. Single CNN, 5. Dual CNN, 6. Quads CNN, 7. Multi-Source Fusion CNN, 8. Hybrid CNN+LSTM, 9. Temporal CNN (TCN), 10. Transformer, 11. GRU. a, b, c: GRF experiment 1, 2, 3 respectively. d, e, f: EMG experiment 1, 2, 3 respectively. g, h, i: Markers experiment 1, 2, 3 respectively. j, k, l: F&M experiment 1, 2, 3 respectively.

The F1 score comparison across models (Fig 9) reveals a clear hierarchy in performance. Classical machine learning models—SVM, LDA, and QDA—achieve moderate results on F&M and Marker data but generally underperform compared to deep learning architectures, particularly for EMG and GRF data. Among the classical models, SVM shows slightly better performance on F&M and Markers, whereas differences for EMG are minimal. In contrast, deep learning models—including Single CNN, Dual CNN, Quads CNN, Multi-Source Fusion CNN, and Hybrid CNN+LSTM—consistently achieve the highest F1 scores across all data types and experiments. Paired t-test results confirm that these differences between classical and deep models are statistically significant (p < 0.05), while differences among top deep models are mostly non-significant, indicating similar levels of excellence (Fig 10).

#### 4.3.2. Experiment-to-experiment analysis.

**Fig 11:**
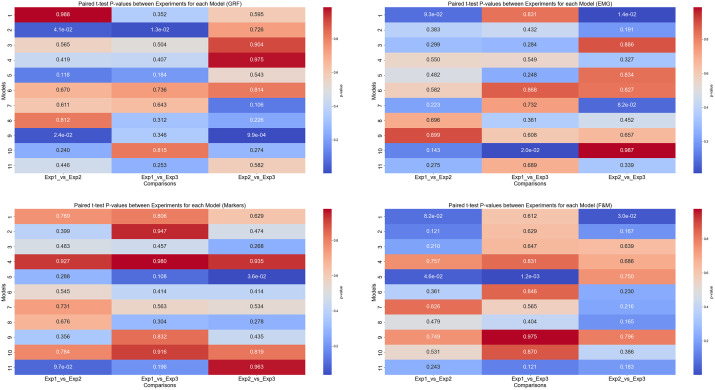
Heatmap values paired t-test and P-values between experiments. For models: 1. SVM, 2. LDA, 3. QDA, 4. Single CNN, 5. Dual CNN, 6. Quads CNN, 7. Multi-Source Fusion CNN, 8. Hybrid CNN+LSTM, 9. Temporal CNN (TCN), 10. Transformer, 11. GRU. a: GRF, b: EMG, c: Markers, d: F&M.

Examining stability across experiments, GRF-based models are more sensitive to changes in experimental conditions, with classical models showing substantial F1 score drops from Experiment 1 to Experiment 2. EMG-based models exhibit moderate performance variability; deep learning architectures maintain relatively high scores, while classical models, especially LDA and QDA, display large fluctuations. Marker and combined F&M data are the most robust across experiments, with minimal F1 score variations for deep models. Paired t-tests between experiments show that performance differences for classical models on EMG and GRF are statistically significant, whereas deep models on Markers or F&M data demonstrate largely non-significant differences, confirming their robustness (Fig 11).

#### 4.3.3. Best model and data.

Considering both overall performance and stability, the Quads CNN is the best-performing model, achieving the highest F1 scores across all experiments (96.6% experiment 1, 96.2% experiment 2, 95.9% experiment 3). The Hybrid CNN+LSTM and Multi-Source Fusion CNN also exhibit consistently high performance, slightly below the Quads CNN model. Regarding data types, Markers alone are the most informative and robust, producing high and stable F1 scores. F&M as second-best data. GRF features perform well with deep models but show higher variability, while EMG contributes less to classical models but remains effective with CNN architectures.

#### 4.3.4. Statistical interpretation.

The paired t-test used in this analysis evaluates whether the mean differences in F1 scores are significant between two paired samples (models or experiments). The t-statistics are computed as:


$$t=\frac{{\bar{X}}_{d}}{s_{d}/\sqrt{n}}$$ (6)

where ${\bar{X}}_{d}$ is the mean difference, $s_{d}$ the standard deviation of the differences, and n the number of paired samples. A p-value < 0.05 indicates a statistically significant difference. The low p-values between classical and deep learning models validate the superior performance of deep architectures, while non-significant p-values among the top deep models suggest comparable excellence.

### 4.5. XAI perturbation

Once the classification task was complete, it became essential to identify not only the most accurate model but also the most effective sensor data source. While multi-source data can provide a richer informational context, wearable sensors often impose practical challenges, such as patient discomfort, making it crucial to determine which data modality yields the most reliable performance.

**Fig 12:**
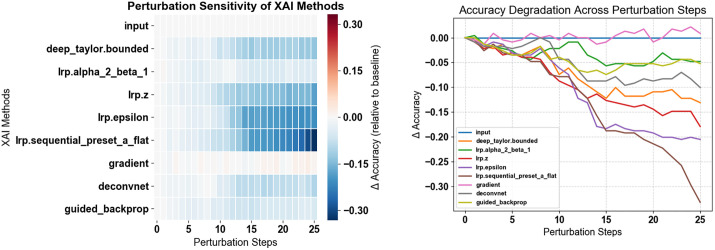
Comparison of XAI Analyzers on Markers: Input, deep Taylor bounded, Deep Taylor, LRP alpha 2 beta 1, LRP alpha 2 beta 1, LRP-Z, LRP-Epsilon, sequential preset a flat, gradient, deconvnet, guided backprop. a: heatmap, b: line plot.

To address this issue, we implemented a perturbation analysis, to select the optimal Layer-wise Relevance Propagation (LRP) method for our study. Fig 12a and b compare several XAI analyzers by examining how classification accuracy declines as the most relevant features are progressively removed. A steeper initial drop in accuracy indicates that the method effectively pinpoints influencing classification in this perturbation process both in the curves and heat maps.

- LRP-Alpha2-Beta1 produced a moderate decrease in accuracy, suggesting that it balances the identification of crucial and less critical features.
- LRP-Z and LRP-Epsilon showed steeper accuracy declines, implying that they focus predominantly on key features, albeit with less overall robustness.
- Sequential Preset-A Flat exhibited the most significant drop, indicating comparatively weaker relevance scoring.
- Methods such as Guided Backpropagation and Gradient, which are not based on LRP, showed slower accuracy degradation, reflecting a more stable feature relevance assignment.

**Fig 13:**
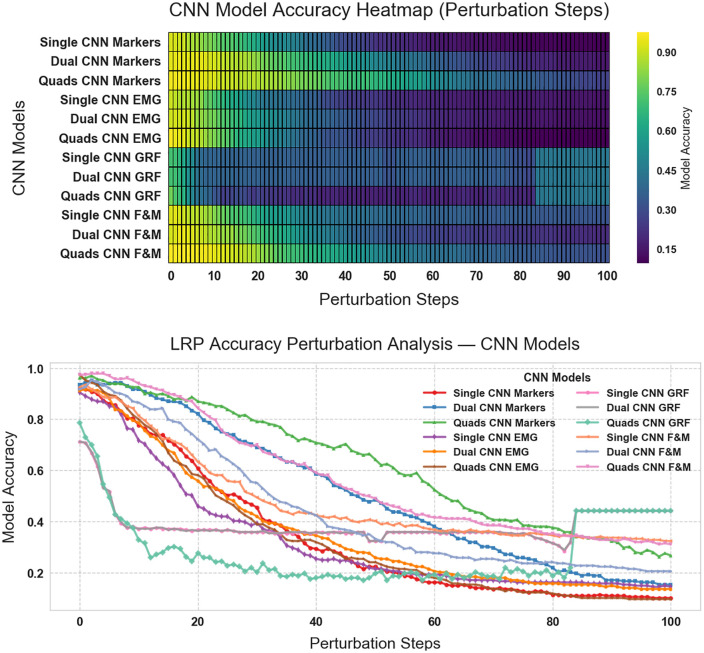
Comparison of CNN models of experiment 1 perturbation over 100 steps for Single CNN, Dual CNN, Quads CNN for Markers, EMG, GRF, and F&M datasets. a: heatmap, b: line plot. With Quads CNN models using Markers data maintain robust performance in the heatmap, b: line plot.

Based on these findings, the Sequential Preset-A Flat method was identified as the most effective LRP variant for our subsequent analysis. Fig 13a and b presents the accuracy analysis curves and heat maps to validate the best model–data pair. This analysis progressively removes the less relevant features and keeping the most important features, and the corresponding drop in classification accuracy is monitored. The results indicate that the Quads CNN models using Markers data maintain robust performance. The gradual decrease in accuracy while using Markers data underscores their resilience and efficacy in capturing the critical features necessary for precise gait classification.

The perturbation analysis underscores the importance of selecting the appropriate LRP method with proper sensor modality. The results suggest that when traditional classifiers provide a useful baseline, the combination of hybrid CNN models like Quads CNN and Dual CNN with carefully selected sensor data yields superior performance in classifying gait speeds. This integrated approach not only ensures high classification accuracy but also enhances the interpretability of the model, making it highly suitable for both engineering applications and clinical use.

### 4.5. XAI explainability analysis

**Fig 14:**
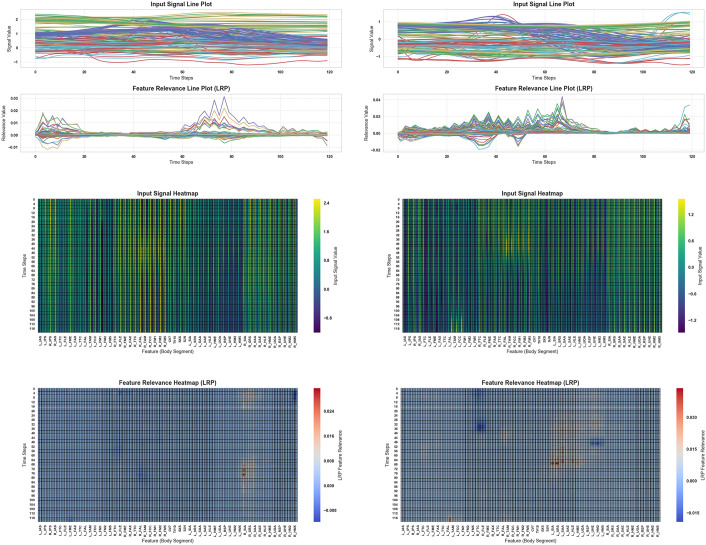
Explainability analysis of the top Quads CNN model from Experiment 2 using LRP (Layer-wise Relevance Propagation) on input samples. (a & b) Top row: original input signals; bottom row: feature relevance maps from LRP. (c & d) Input samples visualized as heatmaps over body segment reflective markers (as defined in Fig 2 and Table 1). (e & f) Corresponding feature relevance heatmaps mapped onto body segment markers. Panels a, c, e corresponds to Class 0 (very slow gait, 0–0.4 m/s), and panels b, d, f corresponds to Class 1 (slow gait, 0.4–0.8 m/s). For Class 0, the most influential features are: Right acromial tip, Right spine root, and right acromial angle coordinates. For Class 1, the most influential features are: Left acromial tip, left spine root, and left acromial angle coordinates.

**Fig 15:**
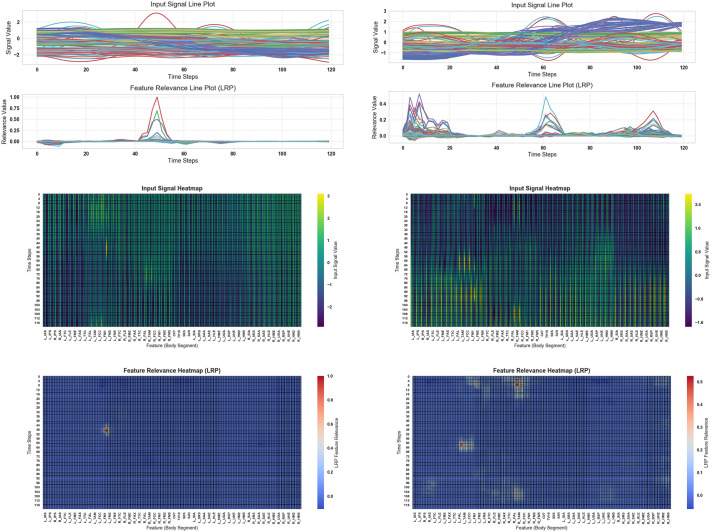
Explainability analysis of the top Quads CNN model from Experiment 2 using LRP (Layer-wise Relevance Propagation) on input samples. (a & b) Top row: original input signals; bottom row: feature relevance maps from LRP. (c & d) Input samples visualized as heatmaps over body segment reflective markers (as defined in Fig 2 and Table 1). (e & f) Corresponding feature relevance heatmaps mapped onto body segment markers. Panels a, c, e corresponds to Class 2 (moderate (0.8–1.2 m/s) or self-selected natural speed), and panels b, d, f corresponds to Class 3 (fast preferred speed). For Class 2, the most influential features are: Left posterior calcaneus coordinates, left 1st metatarsal head coordinates, Left 2nd metatarsal head coordinates. For Class 3, the most influential features are: Left and Right lateral tibial malleolus coordinates, Left and Right medial tibial malleolus coordinates.

To investigate the contributions of specific body segment markers to gait classification, we performed Layer-wise Relevance Propagation (LRP) explainability analysis on the top-performing Quads CNN model from Experiment 2, which was selected based on both subject-splitting across three experimental strategies and perturbation analysis to identify the optimal model. Figs 14 and 15 present the results across different gait speed classes. In Fig 14, panels (a & b) show the original input signals, while the corresponding bottom rows depict feature relevance maps derived from LRP. Panels (c & d) visualize the input samples as heatmaps mapped onto body segment reflective markers, and panels (e & f) illustrate the feature relevance heatmaps for each marker. For the very slow (Class 0, 0–0.4 m/s) and slow (Class 1, 0.4–0.8 m/s) gait classes, the most influential markers were predominantly located on the acromial and spine regions, with Class 0 showing the highest relevance for the right acromial tip, right spine root, and right acromial angle, and Class 1 showing the highest relevance for the corresponding left-side markers. Similarly, Fig 15 shows the explainability analysis for moderate (Class 2, 0.8–1.2 m/s) and fast (Class 3, preferred) gait speeds. Here, Class 2 gait was mainly influenced by the left posterior calcaneus, left 1st metatarsal head, and left 2nd metatarsal head coordinates, while Class 3 gait relevance was concentrated on the bilateral lateral and medial tibial malleolus coordinates. These results highlight how the model selectively leverages different anatomical regions depending on the gait speed, emphasizing the role of upper body markers in slower gaits and lower leg markers in faster gaits.

## 5. Discussion

### 5.1. Overview of findings

The study of human gait speed presents a fascinating intersection of biomechanics, neurology, and artificial intelligence (AI). The integration of convolutional neural networks (CNNs) with Explainable AI (XAI) techniques has enabled a nuanced understanding of gait dynamics, revealing complex interactions between biomechanical processes and neuromuscular control. This research highlights the critical role of hybrid CNN models in accurately classifying gait speeds across a wide range of categories. These models perform exceptionally well in distinguishing extreme speeds, such as very slow and very fast walking, yet they encounter challenges when identifying the more subtle variations inherent in intermediate speeds. The multimodal approach, which leverages data from trajectories, reflective markers, force plates, and electromyography (EMG) sensors, has proven instrumental in achieving high predictive accuracy. In direct comparison, multi-stream Quads CNN have consistently outperformed CNN+LSTM, Single and Dual CNN, TCNs, transformer neural networks, GRU, and statistical classifiers (e.g., Linear Discriminant Analysis, Quadratic Discriminant Analysis, Support Vector Machines), achieving the highest F1 scores across all experiments.

### 5.2. The role of multimodal data fusion

One of the cornerstone achievements of this study lies in the effective use of multimodal data fusion. Traditional single sensor approaches often fall short of capturing the dynamic interplay among these elements. The fusion process allows the CNN models to learn intricate patterns that are difficult to discern from single modalities. For example, reflective marker data provides a detailed three-dimensional view of body movement trajectories, while EMG data reveals the temporal patterns of muscle activation. Combining these sources enables the models to identify correlations between muscle activity and spatial displacement, which are critical for understanding gait dynamics. Moreover, this approach compensates for individual sensor limitations, such as noise in EMG signals or variability in ground reaction force measurements. By systematically replacing the relevant features with noise, this research assesses the resulting decline in accuracy, offering valuable insights into each model’s dependency on specific features for accurate classification.

### 5.3. Hybrid CNN models and classification accuracy

The development of hybrid CNN models, including architectures that combine CNN with Long Short-Term Memory (LSTM) networks, marks a significant advancement in gait analysis. In these hybrid configurations, CNN layers extract spatial features from the multimodal datasets, while LSTM layers capture the temporal dependencies present in gait sequences. This combination is particularly beneficial for analyzing gait speed since the temporal evolution of biomechanical features, such as stride length and joint angles, is a key determinant of walking velocity.

The findings of the work underscore the importance of leveraging deep learning techniques to handle high-dimensional, multimodal datasets and indicate that the hybrid CNN+LSTM architectures are highly effective in capturing both spatial and temporal nuances in human gait.

### 5.4. Explainable AI and interpretability

The incorporation of XAI techniques has added a critical layer of interpretability to this study. While CNNs are highly effective at pattern recognition, their “black box” nature often raises concerns about trust and reliability, especially in clinical applications. By integrating methods such as LRP, we gained valuable insights into the models’ decision-making processes. These techniques revealed the most influential features for gait speed classification, such as muscle activation during the stance phase and variations in joint angles.

The use of LRP, in particular, allowed us to use these heatmaps representing feature relevance. For example, in scenarios involving rapid acceleration, the models emphasized ground reaction forces, whereas slower gait speeds were more strongly influenced by joint angle variations. This enhanced transparency is invaluable for clinicians and researchers, enabling them to validate the model’s findings against established biomechanical principles and to foster greater trust in the predictive capabilities of the system.

### 5.5. Biomechanical and neuromuscular insights

Our findings align with and extend toward existing knowledge in biomechanics and neurology. Muscle activation patterns, especially those captured through EMG signals, emerged as critical determinants of gait speed. The CNN models frequently identified distinct patterns corresponding to specific gait phases, such as the early stance or swing phase, corroborating previous studies on neuromuscular coordination.

Additionally, joint kinematics like notable variations in ankle and knee angles, played a significant role in the classification process. These kinematic variables are crucial for propulsion and stability during walking. Furthermore, our focus on ground reaction forces underscored the interaction between external mechanical forces and internal neuromuscular processes, which is particularly important during transitions such as acceleration and deceleration. Together, these biomechanical and neuromuscular insights provide a robust framework for understanding the determinants of gait speed and pave the way for more targeted interventions in clinical settings.

### 5.6. Implications for clinical practice

The insights gained from this study have significant implications for clinical practice, particularly in rehabilitation and diagnostics. By identifying the most critical features for gait speed classification, this research lays the groundwork for developing targeted interventions. For instance, specific rehabilitation programs could be designed for patients recovering from strokes or lower-limb injuries to enhance specific muscle activation patterns and improve joint mobility.

Additionally, the integration of XAI techniques offers promising opportunities for personalized medicine. Clinicians can use heatmaps generated by LRP to identify areas of dysfunction in a patient’s gait; for example, if reduced relevance is observed in ankle movement, targeted exercises or relevant orthotic devices may be recommended. The high classification accuracy achieved also holds promise for the early detection of neurodegenerative diseases like Parkinson’s, where subtle changes in gait can serve as early clinical indicators.

Beyond clinical applications, the findings of this study have broader implications for fields such as human-robot interaction and autonomous systems. In human-robot collaboration, accurately understanding gait speed is critical for ensuring safety and efficiency; robots can leverage gait predictions to anticipate human movements and adjust their actions accordingly. Similarly, precise gait analysis can improve pedestrian safety in autonomous vehicle systems by enabling more accurate forecasts of walking trajectories.

Furthermore, monitoring the gait speed becomes increasingly important with age for maintaining an independent life. Early detection of mobility decline can lead to timely interventions that prevent falls and preserve functional abilities. The integration of multimodal data and advanced AI techniques, as demonstrated in this paper, thus offers a powerful tool for addressing both technological challenges and broader societal issues.

### 5.7. Limitations and challenges

Despite the promising results, the study is not without limitations. One of the primary challenges is the classification of intermediate gait speeds, which involves subtle biomechanical variations that can be difficult to capture even with advanced models.

Another limitation is the reliance on a publicly available dataset, which may not fully represent the diversity of human gait influenced by factors such as age, weight, and health status. Expanding the dataset to include a broader range of participants would likely enhance model generalizability. Moreover, while the study focused on four distinct speed categories, real-world applications may require a more granular classification, necessitating further model refinement.

### 5.8. Future directions

Based on the findings of this study, several avenues for future research emerge. First, integrating additional data modalities, such as video analysis or inertial measurement units (IMUs), could provide a more comprehensive understanding of gait by capturing aspects not fully represented in the current dataset.

Second, the development of real-time gait analysis systems has an exciting prospect. Deploying hybrid CNN models on edge devices like wearable sensors or smartphones could enable continuous monitoring of gait speed in natural settings.

Additional features, such as cadence variability or stride symmetry, should be explored to improve the accuracy of the classification of intermediate gate speed.

Finally, further exploration of other XAI techniques should be considered as a direction of future work. While methods like LRP and saliency maps have provided valuable insights in this paper, alternative approaches such as counterfactual explanations or attention mechanisms may offer additional perspectives further to enhance model interpretability for clinical and research applications.

## 6. Conclusion

This paper focuses on multimodal data fusion and classification for gait speed predictions using both traditional machine-learning techniques and deep-learning-based CNN models. However, the proposed methods are also applicable for clinical practice to diagnose pathological or monitor cognitive impairment and strokes in elderly patients. Among the classifiers tested in this paper, SVM performed the best among the traditional approaches, with notable F1 scores and high accuracy. However, the hybrid CNN model outperforms SVM scores in gait analysis when trained with the marker data. Through the combination of CNN models and XAI, this study offers both a technical and philosophical exploration of human gait speed. While multimodal data fusion enhances classification accuracy, wearable sensors introduce practical challenges. It has been observed that the marker data is the most effective modality, even under the unstable state of the key model features. The results reveal a deeper understanding of how the human body orchestrates movement and adapts to various internal and external stimuli. By classifying the movement and adapting the critical features contributing to these variations, we advance our comprehension of human locomotion in clinical and everyday contexts. The philosophical questions in the earlier sections—regarding the nature of movement and its relationship to health—are partly addressed through these experimental results, highlighting the intricate and dynamic interplay between body, mind, and motion.
